# Allocentric or Craniocentric Representation of Acoustic Space: An Electrotomography Study Using Mismatch Negativity

**DOI:** 10.1371/journal.pone.0041872

**Published:** 2012-07-27

**Authors:** Christian F. Altmann, Stephan Getzmann, Jörg Lewald

**Affiliations:** 1 Career-Path Promotion Unit for Young Life Scientists, Kyoto University, Kyoto, Japan; 2 Human Brain Research Center, Graduate School of Medicine, Kyoto University, Kyoto, Japan; 3 Department of Cognitive Psychology, Faculty for Psychology, Ruhr University Bochum, Bochum, Germany; 4 Leibniz Research Centre for Working Environment and Human Factors, Dortmund, Germany; University of Salamanca- Institute for Neuroscience of Castille and Leon and Medical School, Spain

## Abstract

The world around us appears stable in spite of our constantly moving head, eyes, and body. How this is achieved by our brain is hardly understood and even less so in the auditory domain. Using electroencephalography and the so-called mismatch negativity, we investigated whether auditory space is encoded in an allocentric (referenced to the environment) or craniocentric representation (referenced to the head). Fourteen subjects were presented with noise bursts from loudspeakers in an anechoic environment. Occasionally, subjects were cued to rotate their heads and a deviant sound burst occurred, that deviated from the preceding standard stimulus either in terms of an allocentric or craniocentric frame of reference. We observed a significant mismatch negativity, i.e., a more negative response to deviants with reference to standard stimuli from about 136 to 188 ms after stimulus onset in the craniocentric deviant condition only. Distributed source modeling with sLORETA revealed an involvement of lateral superior temporal gyrus and inferior parietal lobule in the underlying neural processes. These findings suggested a craniocentric, rather than allocentric, representation of auditory space at the level of the mismatch negativity.

## Introduction

In everyday life, we permanently move our body, head, and eyes while perceiving the environment with our different senses. Thereby, the environment is experienced as remaining stable, and the spatial alignment of information received by different sensory organs is always maintained. From a neuroscientific perspective, this phenomenon of perceptual stability is far from trivial. For example, the position of a light and sound emitting object located in extrapersonal space is simultaneously estimated *(1)* by visual information received by the eyes moving in their orbits (that is, in oculocentric coordinates) and *(2)* by auditory information (namely interaural differences in time and level as well as monaural spectral cues [1]) received by the two ears and thus referenced to the head (that is, in craniocentric coordinates), while both these types of spatial information change with respect to the body, which again moves in the environment.

There is a substantial body of evidence, primarily based on single-neuron studies in various animal species, that auditory and visual spatial information is integrated in the brain (for review, see [2]) and the alignment of sensory coordinates in an oculocentric (eye-centered) frame of reference is maintained with eye movements using an eye-position signal [3]. Moreover, higher-order coordinate transformations have been proposed, resulting in craniocentric (head-referenced), body-referenced, and world-referenced (allocentric) coordinate frames by using neck-proprioceptive and vestibular inputs (for review, see [4]). While auditory-visual spatial integration is known to take place as early as at the level of subcortical structures, namely in superior colliculus [2], the posterior parietal cortex (PPC) has been suggested as the primary locus where the different coordinate frames of the various input signals are combined into common, distributed spatial representations, and where neural activities within these representations are related to higher-order spatial and non-spatial cognitive functions [4].

A crucial problem with models involving sensory inputs on head and/or body position, however, is that almost all neurophysiological work on this topic in humans or non-human species has been conducted with head and body fixation, usually with changes in eye position. Thus any reliable conclusions on representations of sensory space with movements of head and/or body were prevented. Only few studies, employing passive head or body rotation in the monkey, provided direct experimental data on the cellular level indicating that, besides oculocentric spatial representations, craniocentric and allocentric reference frames for sensory stimuli may exist in the PPC (e.g., [5]–[7]; for reviews, see [4], [8]).

In a recent EEG study in human subjects, Altmann et al. [9] investigated the frame of reference of sound localization after horizontal head rotations by measuring the mismatch negativity (MMN) in an oddball paradigm. The MMN is a change-related response that occurs after a series of repeated so-called standard stimuli interrupted by a deviant stimulus. Several EEG studies have described an MMN elicited by auditory spatial changes (e.g. [10]), and that its amplitude depends on the extent of spatial deviance [11]–[13]. Altmann et al. [9] observed a significant MMN for the craniocentric but not for an allocentric deviant after horizontal head rotations. These results argued in favour of a craniocentric representation of auditory space at the level of the MMN. However, later parts of the ERP from about 220 ms after stimulus onset were compatible with both craniocentric and allocentric representations of auditory space. These later components, elicited by either craniocentric or allocentric deviants, resembled in their latency and topography the novelty P3, a positive deflection that can be observed in sequence with the MMN after presentation of an infrequent sound [14].

The study by Altmann et al. [9] simulated auditory lateralization by using generic (artificial head) head-related transfer functions (HRTFs). Thus, it has remained unclear whether these findings apply for the natural situation of a free sound field. Recently, EEG results by Getzmann and Lewald [15] indicated significant differences depending on whether such artificial stimuli or actual sound locations in the free field were presented, suggesting that the latter stimuli allow more reliable conclusions with respect to the neural processing of auditory space.

**Figure 1:**
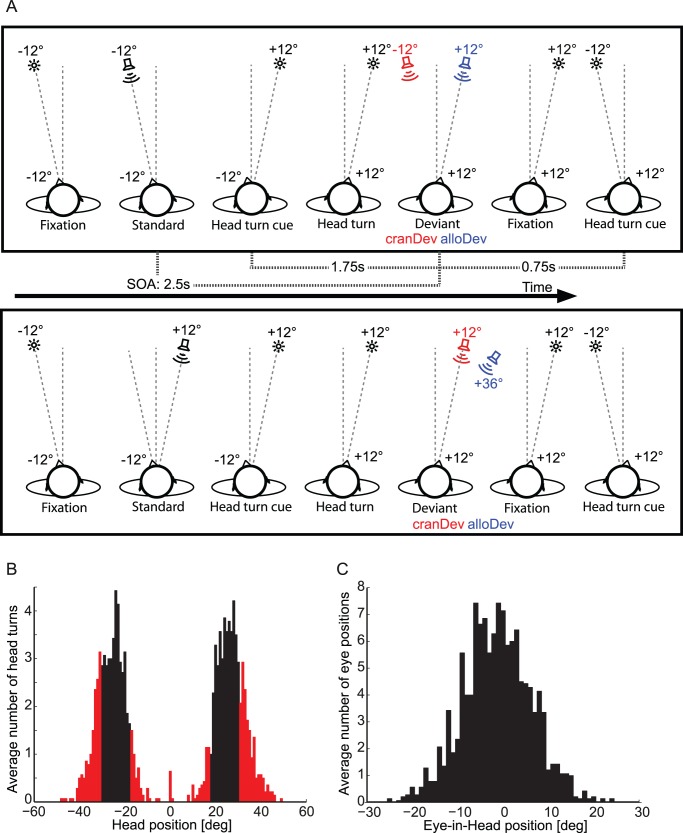
Experimental procedures and behavioral performance. (A) Experimental conditions. The *upper row* illustrates conditions with standard series for which the stimuli were presented in the median plane of the subject’s head, and the *lower row* illustrates conditions with standard series for which stimuli were presented laterally to the median plane of the subject’s head. The standard series (Std) was followed by a deviant (Dev) which was either craniocentric (cranDev, red) or allocentric (alloDev, blue). The angles next to the schematic heads (−12° and +12°) indicate the orientation of the head in relation to trunk median sagittal plane. The angles (−12°, +12°, +36°) adjacent to the loudspeakers and fixation targets (stars) indicate the stimulus positions. Only left to right head movements are shown. In half of the blocks, subjects had to perform right to left head orientations and the stimulation was accordingly mirror-symmetric. (B) Histogram of head positions: mean number of head positions before the deviants, averaged across subjects in one-degree-bins. Black bars indicate trials with head rotations that we deemed sufficiently accurate (within ±6°) to be included into the EEG analysis. Red bars indicate discarded trials. (C) Histogram of eye-in-head positions: mean number of eye-in-head positions at the time of deviant stimulation, averaged across subjects in one-degree-bins.

In the present study, we tested for allocentric versus craniocentric representation of auditory space under free-field conditions. To this end, we recorded the MMN using EEG for noise sequences presented from either a left or a right position (Fig. 1A). Subjects were occasionally cued to rotate their head horizontally at a visual target (with the eyes fixating at the target). After that, they were presented with either a stimulus from the same sound source, which was now a deviant in terms of a craniocentric frame of reference. Alternatively, subjects were presented with a stimulus from a spatially different sound source, which was a deviant in terms of an allocentric frame of reference, but also was a standard in terms of a craniocentric frame of reference. We hypothesized that an MMN should emerge for craniocentric, but not allocentric, deviants, as was shown in Altmann et al. [9].

## Results

### Head Rotation and Eye Position Performance

During the EEG experiment, subjects were instructed to point with their head to a visual fixation target. A histogram of head positions in relation to the fixation target preceding the sound deviants is shown in Figure 1B. A portion of 64.3% of the head rotations were within the desired range of ±6° of the target angle. Figure 1C shows the eye-in-head positions averaged across subjects for all deviants. The average eye-in-head position during deviant presentation was −1.3°±7.5° (SD).

### EEG Results

**Figure 2:**
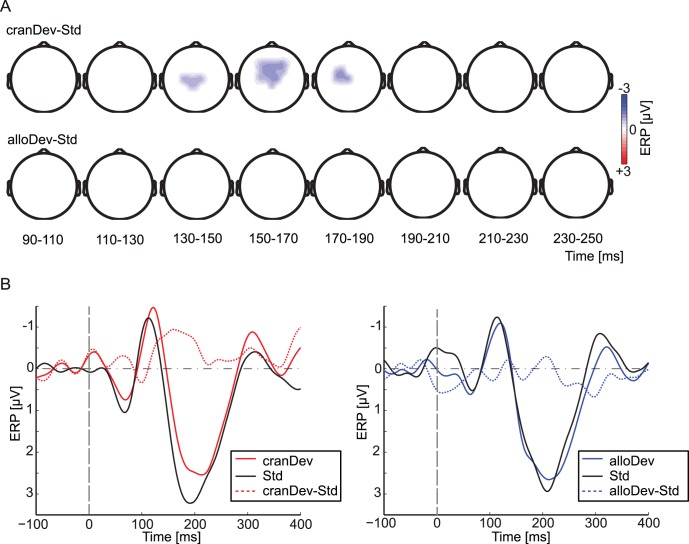
Event-related potentials. (A) Statistical comparisons of craniocentric deviant versus standard and the allocentric deviant versus standard with a nonparametric cluster–randomization approach (two-tailed *t*-test, corrected for multiple comparisons at a cluster-level). Only difference data from electrodes are shown that were part of a significant cluster (*p*<0.05, cluster level) within the indicated period. The upper row shows significant differences between the craniocentric deviant (cranDev) and the standard (Std), and the lower row differences between the allocentric deviant (alloDev) and the standard. (B) ERP waveforms at fronto-central electrodes for the craniocentric (cranDev, left column) and allocentric deviants (alloDev, right column), and for standards (Std). Solid colored lines indicate ERPs for the deviant (red: craniocentric; blue: allocentric), black lines the standard, and dashed colored lines the difference waves between craniocentric (red) or allocentric (blue) deviants and standards. The vertical dashed gray lines indicate stimulus onset.

We statistically compared the ERPs for deviant and standard stimuli in all the conditions using a cluster-based randomization statistics (Figure 2A). Significant fronto-central negativity (*p*<0.05 at a cluster level) for deviants compared to standards was observed for the craniocentric deviant only, between 136 and 188 ms after stimulus onset. This negativity was centered around the FCz and Cz electrodes. There was no significant positivity for this condition. No statistically significant differences between deviants and standards were obtained in the allocentric condition.

Figure 2B depicts the ERP time-course from fronto-central electrodes averaged across all subjects. We calculated the MMN amplitudes as the mean difference between deviants and standards in the time interval of 100–250 ms after stimulus onset, the time window in which the MMN is typically observed (e.g., [10]). These mean amplitudes were on average −1.01±1.31 µV (SD) for the craniocentric and 0.10±1.28 µV for the allocentric deviant. A paired-sample two-tailed *t*-test revealed significantly more negative amplitudes for the craniocentric, than to the allocentric, MMN (*t* = 2.49, *p*<0.05). The MMN peak latencies were on average 182±39 ms (SD) for the craniocentric and 181±43 ms for the allocentric deviant-standard difference curve.

**Figure 3:**
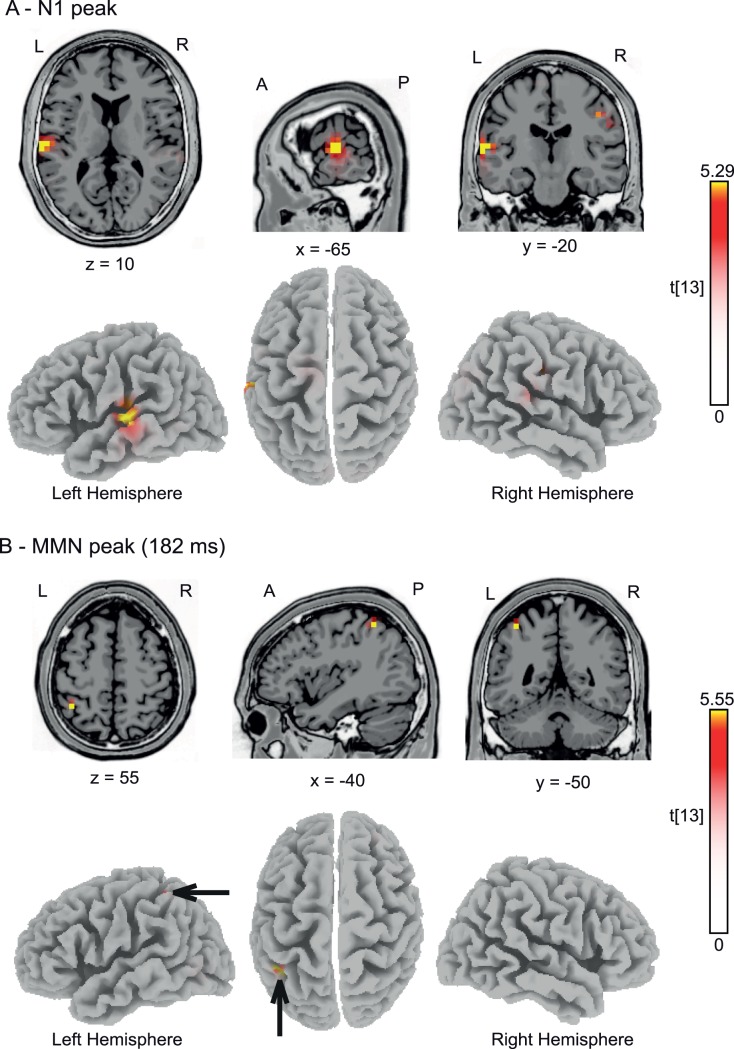
Source localization. (A) sLORETA source reconstruction for the N1 component of the craniocentric deviants versus standards (measured in trials preceding the craniocentric deviants). Functional data from all subjects were projected onto the standard 3-D MNI brain template “Colin” of sLORETA. Red and yellow color coding indicates *t*-values with statistically significant activation for deviants compared to standards at *t*≥5.29; *p*<0.05, corrected for multiple comparisons. (B) sLORETA source reconstruction for the craniocentric deviant compared to standards for time points with maximal MMN (mean: 182 ms). Red and yellow color coding indicates *t*-values with statistically significant activation for deviants compared to standards (*t*≥5.55; *p*<0.05, corrected for multiple comparisons). Black arrows point toward significant regions in the left inferior parietal lobule. A, anterior; P, posterior; L, left; R, right. The *x*, *y*, and *z*-values indicate the position of the sagittal, coronal and horizontal slices in MNI-coordinates.

To characterize the underlying neural generators of the MMN, we conducted sLORETA source reconstruction for two different time frames: firstly, we statistically compared the N1 responses to craniocentric deviants to the standards immediately preceding the craniocentric deviants. The rationale to evaluate the N1 time range was that previous studies have suggested that N1 attenuation due to stimulus repetition may contribute to MMN generation [16]. We determined the peak N1 latencies for individual subjects and separately for the deviant (119±15 ms) and standard (109±16 ms) conditions. We then calculated the voxel-based sLORETA-images of the ERPs in a 10-ms time window around these latencies for deviant and standard stimuli, and submitted these images to statistical tests, using the sLORETA-built-in voxelwise randomization test (5000 permutations) based on statistical non-parametric mapping (SnPM), corrected for multiple comparisons. Significantly (*t*≥5.29, *p*<0.05) activated voxels are shown in Figure 3A and were located in the left lateral superior temporal gyrus (MNI coordinates: *x* = −65 mm, *y* = −20 mm, *z* = 10 mm). Secondly, we determined the peak MMN latencies at the time points of maximal difference between craniocentric deviant and standards for individual subjects, and conducted a similar sLORETA analysis. We observed one single area in left inferior parietal lobule (IPL, Brodmann area 40; MNI coordinates: *x* = −40 mm, *y* = −50 mm, *z* = 55 mm; Fig. 3B) showing significantly (*t*≥5.55, *p*<0.05) more intense activation for deviants than standards.

## Discussion

The present study investigated the MMN related to changes in sound location and head position, with the primary focus on the type of representation of auditory space and on the cortical substrate of this representation. As in a previous study [9], we observed a significant MMN exclusively for the craniocentric deviant, suggesting a primarily craniocentric representation at the level of the MMN. This finding extends the earlier results insofar as the present method of auditory stimulation, employed in the free sound field under carefully controlled anechoic conditions, may exclude any potential artifacts associated with the previously used presentation of sound via headphones (for a detailed discussion of this issue, see [15]). Most importantly, the sLORETA distributed source model revealed areas in the lateral superior temporal gyrus and the IPL as the generators for the craniocentric change-related EEG response.

Previous studies eliciting responses related to auditory spatial change have stressed the importance of superior temporal areas in the generation of this signal. The study by Deouell et al. [12] localized spatial MMN generators in the posterior superior temporal lobe employing LORETA. A later series of fMRI experiments confirmed these findings and described sensitivity of the medial planum temporale (PT) to auditory spatial changes [17]. This is in line with fMRI studies that contrasted pitch and spatial changes and found in particular an involvement of the posterior medial PT in the processing of spatial change [18]. In the present EEG study, the difference between craniocentric deviants and standards at the time of the N1 component accordingly showed localization in lateral superior temporal gyrus. Later, at the time of the maximal difference between the craniocentric deviants and standards (on average 182 ms after stimulus onset), neural generators were localized within IPL. An involvement of the IPL in the processing of auditory spatial changes is generally in line with models that proposed a posterodorsal cortical pathway to preferentially underlie auditory spatial processing beyond primary auditory cortex in the primate brain [19], [20]. This proposed “where” stream includes posterior superior temporal cortex, PPC, and dorsolateral prefrontal cortex. A central role of the human IPL in the processing of spatial sound attributes has been described in several studies, employing fMRI, positron emission tomography, magnetoencephalography, and EEG (e.g., [21]–[32]). That activation was found here in the left hemisphere, might be in partial opposition to previous investigations that mostly suggested a dominance of the right hemisphere for auditory spatial processing in parietal lobe [22]–[24], [27], [33], [34]. On the other hand, in their meta-analysis of imaging studies, Arnott et al. [28] concluded that there did not appear to be any hemispheric lateralization for spatial processing tasks, with even a numerical tendency of more frequent reports of unilateral left-hemisphere, rather than right-hemisphere, activity (30% vs. 20%; 50% bilateral) as may be in alignment with our data.

It has been proposed that the parietal cortex may subserve the neural transformation of the coordinates of the auditory space in order to generate body- or world-referenced (allocentric) spatial coding [34]. This idea has been derived from studies on visual neurons in the intraparietal sulcus of the monkey, which is part of the monkey homologue of human PPC [5]–[7], [35]. A portion of these visual neurons exhibited a spatial selectivity also for auditory stimuli in a manner such that auditory and visual receptive fields were aligned [36], [37] (for review, see [4]). The PPC may thus represent a main site of multisensory integration, in particular performing coordinate transformations to align the spatial information from different sensory modalities [4], [38]. In detail, in macaque intraparietal cortex a combination or “hybrid” of eye- and head-centered frames of reference for encoding visual and auditory locations has been found in experiments in which head-, body- and world-centered reference frames were all stable with respect to each other [37], [39], [40]. More important in the context of the present work is the finding of a body-centered map of visual space, formed by the modulation of retinal responses by gain fields to gaze position, within the intraparietal sulcus, as was revealed by variation of the monkey’s head-to-trunk position during neurophysiological recordings [6], [7]. Using fMRI in human subjects, Brotchie et al. [41] demonstrated properties very similar to this area of the monkey in a region of human intraparietal sulcus. In particular, the amount of signal change seen in this region was modulated by head position relative to the body, thus suggesting a gain field dependent body-centered representation of space in the human PPC. Whether or not these conclusions can be applied to the auditory modality has until now remained unclear.

If one accounts for the relatively low spatial resolution of sLORETA, the locus of activation revealed here in IPL could actually be identical with the human intraparietal region (parietal eye field) reported by Brotchie et al. [41]. Assuming that this localization holds true, the present activation may be compatible with the previous findings in the visual modality that suggested a combination of eye- and head-centered frames of reference representation of space in intraparietal sulcus [37], [39], [40]. In this context, it has to be emphasized that due to the focus of our approach head- and eye-centered reference frames remained always stable with respect to each other. Thus, any conclusions on a potential transformation of auditory space into an oculocentric frame of reference are not possible, and the present findings can be interpreted by either assuming pure craniocentric, pure oculocentric, or any intermediate coding at the level of the MMN. In each case, our data did not provide any hint for a body/world-centered reference frame for auditory space coding in IPL. Given the low spatial resolution of sLORETA, it has, however, to be noted that it is also possible that the IPL activation could rather represent a genuine part of the posterior parietal level of the posterodorsal auditory pathway, as has been identified by neuroimaging with head fixation (see above). Insofar, one might also conclude that, at this level of the posterodorsal pathway, a transformation of the originally head-referenced auditory coordinates, received by the two ears, into a different reference frame did not occur. To clarify this issue, future studies may include changes of eye-in-head position in addition to head-to-trunk position.

The generating process of the MMN has been a matter of debate for decades (for spatial deviance, see [10], [42]). In particular, two mechanisms have been proposed to result in deviance related activity: a sensory mechanism based on the refractoriness of stimulus-feature-specific neurons (e.g., [16], [43], [44]) and a more cognitive mechanism involving memory-based comparison (e.g., [45]–[47]). In the context of the present study, one could argue that the craniocentric MMN may comprise both mechanisms while the allocentric only involves a memory-based MMN mechanism. This could explain the stronger MMN for the craniocentric compared to the allocentric deviant. To disambiguate the two mechanisms previous studies [10], [47]–[49] have employed a control condition in which stimuli serving as a deviant in the oddball condition were now grouped with different stimuli varying along one stimulus dimension. Applied to the present experiment, the oddball stimulation could consist of series of three standard sounds (e.g., −12°) followed by a deviant (e.g., +12°). A control block could consist of randomly alternating sounds presented with equal probability (e.g., −36°, −12°, +12°, and +36°). As the stimulus presentation probability is the same in the oddball and control conditions, the difference between the deviant ERP of the oddball and the control condition reflects the contribution of a memory-based mechanism to the MMN. Future studies could utilize a similar approach to disentangle the contribution of refractoriness and memory-based comparison to the generation of the craniocentric deviant.

In a previous study [9], a significant positivity from about 220 ms after stimulus onset for deviants compared to standards was observed that resembled a novelty P3 [14]. In contrast, in the present experiment the cluster-based randomization statistics did not reveal a significant novelty P3 in any of the conditions. Possibly, the deviant angles employed (±24°) were too small to generate this novelty P3-like response. Earlier studies investigating the MMN to spatial deviants showed only small positive deflections at around 200 to 260 ms to small deviance angles [13]. Moreover, unlike Altmann et al. [9], sound stimuli were here presented in the free-field. Earlier studies that compared spatial MMN to spatial deviants between head-phone and free-field conditions found a significant P3a mainly in the head-phone condition and in the free-field only for extreme spatial deviance of 90° [11]. To clarify whether the generation of a novelty P3-like component after head rotations depends on the extent of deviance, future studies should employ larger deviants (>30°).

### Conclusions

The results of our study argue in favour of a representation of sound sources in a craniocentric, rather than an allocentric, reference frame at the level of the mismatch negativity from about 136 to 188 ms after stimulus onset. Distributed source modeling with sLORETA indicated the involvement of the left lateral superior temporal gyrus and left IPL (Brodmann area 40) in the generation of these mismatch signals.

## Materials and Methods

### Participants

Fourteen healthy volunteers participated in the EEG experiment (8 females; mean age 27.4 years; range 20–51 years; all right-handed). All listeners had normal hearing tested by pure tone audiometry (HL <20 dB; Oscilla USB100, Inmedico, Lystrup, Denmark) as well as no history of psychiatric or neurological illness. The subjects’ vision was either normal or corrected to normal by spectacles or contact lenses. All subjects gave their written informed consent to participate in the study. The experiment was conducted in accordance with the ethical standards laid down in the 1964 Declaration of Helsinki (sixth revision, 2008) and was approved by the local ethical committee of the Leibniz Research Center for Working Environment and Human Factors, Dortmund, Germany.

### Apparatus and Stimuli

The experiment was conducted in a dimly lit, sound-proof and anechoic room (4.4 m wide ×5.4 m long ×2.1 m high), insulated by 40 cm (height) ×40 cm (depth) ×15 cm (width at base) fiberglass wedges on each of its six sides. The ambient background noise level was below 20 dB(A) SPL. Subjects sat on a vertically adjustable chair. The subject’s head was fixed with a custom-made framework with stabilizing rests for the chin, forehead, and occiput. This head restraint was swivel-mounted in a way such that the head could be freely rotated in the azimuthal plane around the center of the subject’s interaural distance. The azimuthal position of the head was measured by a potentiometer linked to the pivot of the rotating head restraint [50].

Free-field sound stimuli were presented from four broad-band loudspeakers (SC 5.9; Visaton, Haan, Germany; 5×9 cm^2^) that were mounted in the subject’s horizontal plane with a distance of 1.5 m from the center of the head. Loudspeakers were located at −36° (left), −12°, +12° (right), and +36° azimuth with reference to the subjects’ trunk median sagittal plane, at ear level. All loudspeakers were selected on the basis of similar efficiency and frequency response curves. The loud-speaker set-up was part of larger array of loud speakers, as described previously [33], [34]. As cues for head rotations and as fixation targets, two red LEDs (3 mm; 0.025 mcd) were located at the lower edge of the chassis of each loudspeaker −12° and +12°. Head orientation was measured by the potentiometer at the time of each stimulus onset. The timing of the stimuli and the recording of the subject’s head position were controlled by custom-written software. In addition, eye and head positions were monitored online by the experimenter via two infrared video cameras.

The sound stimuli were generated digitally using CoolEdit 2000 (Syntrillium Software Co., Phoenix, AZ, USA). They consisted of continuous, band-pass-filtered (lower and upper cut-off frequencies 0.7 and 3 kHz, respectively) white noise with rise/decay times of 20 ms and a duration of 100 ms. All sounds were presented at 68 dB(A) SPL, measured at the position of the center of the subject’s head.

### Procedure

Before the EEG experiment started, subjects were administered a training block for aligning head orientation and a calibration block for eye position measurement. In particular, subjects repeatedly performed horizontal saccadic eye movements to the two red LEDs (12° left and right). This allowed the estimation of the subjects’ horizontal eye position in visual angle by a linear transformation of the EEG signal acquired at electrode positions LO1 and LO2. After that, twelve of the fourteen subjects completed eight blocks of stimulus presentation during which EEG was recorded. One subject completed six and another subject seven experimental blocks. An experimental block comprised 72 auditory stimuli that were presented at a rate of 0.4 s^−1^ (i.e., one stimulus every 2.5 s.) We used an oddball design to induce MMN in which each standard was presented in sequences of three or four repeated presentations. During the whole experiment, subjects were instructed to keep their eyes open, fixate on the red LED, and align the head to this fixation LED (i.e., point with their nose to the red light) as long as it was on. Furthermore, subjects were explicitly instructed to ignore the auditory stimuli.

Within each experimental block, the craniocentric and allocentric deviants were presented randomly intermixed. During four experimental blocks, subjects had to move from a left standard position to the right before the deviant. In the other four blocks, the subjects had to move from right (standard) to left (deviant). Blocks with left-to-right and right-to-left changes were alternating, with balanced succession across subjects. After each standard sequence, the LED at the standard head position (−12° or +12°) was turned off (750 ms after sound onset of the last standard). Simultaneously, the red fixation LED at the other position (+12° or −12°, respectively) was switched on, and the subjects had to orient their heads to the new fixation target. After the LED position change (1750 ms after target onset), one of two types of spatial deviants was presented: (1) either the deviant stimulus was presented from the same location in space as the preceding standard while the head position changed - this was a deviant in terms of a craniocentric reference frame (cranDev); or (2) the deviant stimulus was presented at a location that compensated for the head rotation, that is, at the identical location with reference to the head, but at a displaced position with reference to external space - this was a deviant in terms of an allocentric reference frame (alloDev). In total, the eight blocks provided 128 changes (i.e., 64 deviants per deviant condition). The standards were defined as the last stimulus of the standard series before occurrence of a deviant.

### EEG Acquisition and Data Analysis

The continuous EEG was sampled at 500 Hz using 57 Ag/AgCl electrodes (referenced to a vertex electrode at FCz) and two cascaded NuAmps amplifiers (NeuroScan Labs, Sterling, VA, USA). Electrode positions were based on the International 10–10 system (AF3/4, AF7/8, AFz, C1–C6, CP1–CP6, CPz, Cz, F3/4, F7/8, FC1–FC6, FCz, FP1/2, FPz, FT9/10, Fz, O1/2, Oz, P1–P4, P7/8, PO3/4, PO7/8, PO9/10, POz, Pz, T7/8, TP7/8). Horizontal EOGs were recorded from two electrodes placed at the cheek bone, toward the outer canthi of each eye. Vertical EOGs were recorded from four electrodes placed above the eyebrows and at the cheek, immediately above and below the centers of the right and left eyes. The ground electrode was placed on the center of the forehead, just above the nasion. Two additional electrodes were placed on the left and right mastoids. Electrode impedance was kept below 5 kΩ. EEG data were analyzed with the BESA software package (MEGIS Software, Gräfelfing, Germany), custom-written Matlab software (The Mathworks Inc., Natick, MA, USA), and the FieldTrip Matlab toolbox (http://fieldtrip.fcdonders.nl/). Before signal averaging, eye-blink artifacts were identified by principal component analysis (PCA) and their effect was removed from the raw data [51]. The remaining artifactual epochs were discarded based on a thresholding procedure which removed epochs with a peak-to-peak amplitude exceeding 120 µV and a slew rate exceeding 120 µV/ms. Trials in which the participants missed the desired head orientation by more than 6° were discarded from analysis. On average, 56.9% of allocentric deviant, 53.7% of craniocentric deviant, and 57.3% of standard epochs were retained after EEG artifact rejection and rejection of epochs with inadequate head position. The raw data were high-pass filtered off-line with a cut-off frequency of 0.5 Hz (slope: 48 dB/oct), re-referenced to the average of 59 channels (57 EEG and 2 mastoid electrodes), and segmented into 800 ms stimulus-locked epochs covering the period from −200 to 600 ms relative to sound onset. ERP data were low-pass filtered with a cutoff frequency of 25 Hz.

For group-level statistical analysis of the ERP and to address the problem of multiple comparisons (200 time points, 57 electrodes), we used cluster randomization analysis described in previous reports [9], [52], [53]. We compared the deviants with standards using a two-tailed Student’s *t*-test in the time window of 50–350 ms after stimulus onset. Clusters were restricted to a minimum size of three neighboring electrodes showing significant differences between conditions (*p*<0.05). As a test-statistic, the sum of *t*-values across a cluster was compared to the distribution of maximum cluster sums of *t*-values derived from a randomization procedure (1000 randomizations). Differences were reported as statistically significant when the cluster *p*-value was below an *α*-value of 0.05. To display the ERP time courses and to assess the MMN in the different conditions, we averaged the EEG signal across the fronto-central electrodes Fz, F3/4, FCz, FC1–4, Cz, and C1–4.

To reconstruct the underlying neural generators of the MMN, we used sLORETA [54]. sLORETA accounts for scalp-recorded electrical fields by dividing the brain into a three-dimensional grid of points and determining a pattern of electrical activity across these points that gives rise to the electrical fields observed at the scalp. Distributed source analysis methods like sLORETA avoid the problem of having to specify the number of sources in advance, as is the case with, e.g., the equivalent dipole analysis method. sLORETA reduces the number of possible solutions by selecting the smoothest distribution of activity, under the assumption that the activities of neighboring brain regions are correlated. sLORETA is a new version of the previous LORETA method [55], [56]. The main difference is that sources are estimated on the basis of standardized current density allowing more precise source localization than LORETA [54]. sLORETA calculates the standardized current density at each of 6239 voxels in the grey matter and the hippocampus of the Montreal Neurological Institute (MNI) brain template [57]. This calculation is based upon a linear weighted sum of the scalp electric potentials (for details of this methodology, see [54]). The electric potential lead-fields were based on a standardized 3-component boundary element method (BEM) model derived from the averaged MNI brain anatomy as described in [58] and implemented in the sLORETA software. The electrode positions were localized according to the 10/10 electrode system and co-registered to the standard head model according to the electrode positions described in [59]. Previous studies have shown reliable localization of possible cerebral sources with sLORETA [60], [61]. We performed sLORETA on the ERPs of the craniocentric deviant and standards for two time windows: firstly, within a 10-ms time-window centered around the individual peak N1 responses derived from fronto-central electrodes (Fz, F3/4, FCz, FC1–4, Cz, and C1–4); and secondly, within a 10-ms time-window centered around the individual peak MMN derived from the same fronto-central electrodes. The resulting deviant sLORETA images were statistically compared with the sLORETA images of the craniocentric standards, using the sLORETA-built-in voxelwise randomization tests (one-tailed *t*-statistic on log-transformed data). Significantly activated areas were defined as voxels that exhibited a *p*-value <0.05, corrected for multiple comparisons by the sLORETA-built-in voxelwise randomization test.
